# Œdema Glottidis Following the Epizooty

**Published:** 1881-01

**Authors:** William Henry Porter, William Soula


### CASE DEPARTMENT.

### I.—A CASE OF ŒDEMA GLOTTIDIS FOLLOWING THE "EPIZOOTIC" CATARRH.

A GREY gelding, æt. fourteen; had coughed for eighteen months prior to death. At frequent intervals during the past eighteen months the horse was noticed coughing up frothy mucus tinged with blood; the cough at other times was dry. These attacks of coughing up bloody sputa always followed severe exercise. The horse had nearly recovered from the "epizoot," and had been able to work up to two days before his death. Two days before death there was an increased frothy discharge from the nostrils, accompanied by cough and hiccoughing. This cough and discharge from the nose gradually increased. The discharge from the nostrils grew thicker and of a yellow coler, but at no time contained any blood. There was some increased heat around the throat, but no marked external swelling. On physical examination, sharp, crackling râles were detected, indicating an œdematous condition of the lungs. The respirations were twenty-eight to the minute. The limbs were cold to the hand, also the whole length of the trachea; urine, so far as noticed, normal; bowels regular. For the last day the horse could not be got to eat. The horse dropped dead in the night. A necropsy was made by Dr. Porter some twelve hours after death. Rigor mortis was well marked; animal in fair flesh. Thoracic cavity:—The pericardial sac contained considerable more fluid than normal. The heart was enlarged and the left ventricle hypertrophied. There was a marked thickening and fatty change both in the ventral and aortic valves, both being insufficient; the lesion being most marked in the ventral. The base of each lung was deeply congested and in a condition known as "splenization." The upper lobes were somewhat congested and œdematous; as a result of this condition, the breathing space was greatly diminished. The lower part of the trachea and bronchia was filled with frothy mucus. The trachea and larynx were both laid open throughout their whole extent. When the upper end of the trachea was reached it was found deeply congested and covered by numerous ecchymotic spots; in the larynx the congestion and echymosis was even more marked, with marked œdema of the aryteno-epiglotidian folds, indicating the condition known as œdema glottis. In the abdominal cavity all the organs appeared normal. The cause of death was undoubtedly œdema glottidis complicated by chronic heart and lung disease.

This case presents points of interest which have not been as fully appreciated as they ought to be. The subject of œdema glottidis and the rapidity with which it produces death, can never be too frequently called to mind. It is a well-known fact, however, that the condition known as œdema glottidis is absolutely incompatible with life. Had tracheotomy been performed the night before, this horse would in all probability have been alive to-day. As there is no special objection to the operation, it ought in veterinary practice to be resorted to early in all cases of severe laryngeal disease. By the early performance of tracheotomy many a valuable horse could be saved. The cardiac lesions are also of great interest, this case proving conclusively that cardiac murmurs, indicating cardiac disease, do occur in horses as well as in human beings. Although these lesions were not detected before death, there is no doubt that they might have been recognized then. But physical diagnosis is another subject that is neglected in veterinary medicine. Had this condition of valvular insufficiency been detected, there is reason to believe that, by a careful administration of remedies, œdema glottidis might have been warded off. From the simple fact that the air space was diminished and the demand for air greater than normal, there was of necessity increased irritation of the laryngeal mucus membrane, and with an impeded blood current, everything was favorable to the development of œdema glottidis.

WILLIAM HENRY PORTER, M. D., and WILLIAM SOULA.

